# SERVICE PROVIDER PERSPECTIVES ON ADVANCE CARE PLANNING USE IN RURAL DEMENTIA PATIENTS AND CAREGIVERS

**DOI:** 10.1093/geroni/igad104.2369

**Published:** 2023-12-21

**Authors:** Peiyuan Zhang, Ebow Nketsiah, Hyunjin Noh

**Affiliations:** University of Maryland, Baltimore, Baltimore, Maryland, United States; Saint Louis University, St. Louis, Missouri, United States; University of Alabama, Tuscaloosa, Alabama, United States

## Abstract

Compared to urban areas, advance care planning (ACP) utilization remains very limited in rural communities. ACP earlier in the disease trajectory is particularly important for people with dementia (PWD) due to its progressive nature affecting their decision-making ability. Considering well-documented benefits of ACP in improving quality of end-of-life (EOL) care, the rural vs. urban disparity may indicate poorer EOL quality for rural PWD. Moreover, research on factors affecting ACP use among PWD has been primarily based in metropolitan/urban areas. To address the knowledge gap, this study aimed to explore barriers and current resources for ACP of PWD from perspectives of health and social service providers in rural Alabama. Using a qualitative approach, semi-structured face-to-face interviews were conducted with 10 health and social service professionals serving older adults and their caregivers in rural communities of Alabama. Thematic analysis was used to find recurrent themes and patterns from transcribed interview data. Our analysis revealed four areas of barriers to rural PWD’s ACP: (1) PWD’s and caregivers’ lack of knowledge about ACP, dementia, EOL care options, and available resources, (2) misconceptions of completing formal documentations, (3) emotional barriers, and (4) limited access to existing resources. Elder law clinic and local Area Agencies on Aging were the most prominent, existing resources for ACP in rural Alabama. Participants also showed a type of misconception that a lawyer and/or a notary is required for ACP. The study highlighted an urgent need for social policy in ACP education for both caregivers and service providers in rural settings.

